# Longer exposure to a new refugee food ration is associated with reduced prevalence of small for gestational age: results from 2 cross-sectional surveys on the Thailand-Myanmar border^1^,^2^,^3^

**DOI:** 10.3945/ajcn.116.148262

**Published:** 2017-05-10

**Authors:** Verena I Carrara, Wolfgang Stuetz, Sue J Lee, Kanlaya Sriprawat, Basi Po, Borimas Hanboonkunupakarn, François H Nosten, Rose McGready

**Affiliations:** 4 Shoklo Malaria Research Unit, Mahidol-Oxford Tropical Medicine Research Unit, Faculty of Tropical Medicine, Mahidol University, Mae Sot, Thailand;; 5 Institute of Biological Chemistry and Nutrition, University of Hohenheim, Stuttgart, Germany;; 6 Department of Clinical Tropical Medicine and; 7 Mahidol-Oxford Tropical Medicine Research Unit, Faculty of Tropical Medicine, Mahidol University, Bangkok, Thailand; and; 8 Centre for Tropical Medicine, Nuffield Department of Medicine, University of Oxford, Oxford, United Kingdom

**Keywords:** birth weight, birth length, head circumference, preconception, pregnancy, preterm birth, ration, refugees, small for gestational age, fetal growth

## Abstract

**Background:** Despite the high risk of compromised nutrition, evidence of the effect of refugee rations on fetal growth is limited. A new ration containing micronutrient-fortified flour without increased caloric content of the general food basket was introduced to the Maela refugee camp in Thailand, July 2004.

**Objective:** The effect of the length of gestational exposure of the new ration on fetal growth was compared with birth outcomes [small for gestational age (SGA), preterm birth (PTB)].

**Design:** In an observational study in 987 newborns from 1048 prospectively followed antenatal clinic (ANC) attendees enrolled in 2 cross-sectional surveys, exposure was categorized in 2004 according to gestation at the time of commencing the new ration and in 2006 as comprehensive (preconception and pregnancy). In both surveys, the pregnancy-specific ration and vitamin supplements were routine.

**Results:** In 2004, the proportions of SGA decreased with longer exposure to the new ration: no exposure during pregnancy (27.7%; *n* = 13 of 47) and exposure in the third (27.6%;* n* = 37 of 134), second (18.6%;* n* = 35 of 188), and first (19.4%;* n* = 6 of 31) trimesters, respectively (adjusted *P*-trend* =* 0.046). In 2006, the new ration was available to all women and there was no significant additional impact of the pregnancy-specific ration and vitamin supplements. Between 2004 and 2006, SGA decreased from 28.9% (13 of 45) to 17.3% (69 of 398) (adjusted *P =* 0.050), a reduction of 40.1% (95% CI: 34.7%, 45.9%); there was also a decrease in the percentage of underweight women on admission to the ANC (38.2%; 95% CI: 31.4%, 45.5%). PTB rates were low and not significantly different with exposure to the new ration.

**Conclusions:** In 2004, the earlier in gestation in which the new ration was available the greater the effect on fetal growth as shown by a reduced prevalence of SGA. In 2006, additional benefits to fetal growth from the pregnancy-specific ration and vitamin supplements beyond those of the preconception ration were not observed. Good nutrition in pregnancy remains an important challenge for refugee populations. This trial was registered at http://drks-neu.uniklinik-freiburg.de/drks_web/ as DRKS00007736.

## INTRODUCTION

Refugee populations are vulnerable in terms of available nutrients, and pregnant women may not be replete in circulating micronutrients (1). Maternal caloric intake and micronutrient status are important determinants of neonatal outcome (2–4). The burden of neonatal mortality and morbidity resulting from being born small for gestational age (SGA)^9^ (5) or having a preterm birth (PTB) (6) is highest in low- and middle-income countries. The major long-term problems of being born SGA relate to an increased risk of noncommunicable diseases (5); for PTB, there is an increased risk of disability and morbidity, which goes beyond early infancy and extends into adulthood in some survivors (7).

Before publication of the Intergrowth-21st international standards for newborn anthropometric measures (8), an evaluation of >10,000 births of refugees and migrants from the Thailand-Myanmar border (2001–2010) reported that 28.9% and 21.2% of neonates were born SGA and 9.2% and 7.5% were born with PTB in women with and without malaria, respectively, during pregnancy (9). These marginalized populations have a relatively high rate of SGA [≤40% categorized as SGA in South Asia (10)] and a low rate of PTB [PTB was estimated at 14% for South East Asia (11)]. Factors reported to be associated with SGA in Myanmar refugee and migrant women included maternal malaria, hypertension, smoking, first pregnancy, having a low BMI, and female sex of the newborn (9). Specific nutritional deficiencies in pregnant women have been reported from this area and include thiamin (12, 13), α-tocopherol and β-carotene (14), as well as anemia, including iron deficiency (15), despite supplementation (16). Recognition of these deficiencies resulted in systematic vitamin supplementation of women at antenatal clinics (ANCs). A recent report suggests high rates of compliance to thiamin and folic acid supplements, reflected in thiamin diphosphate and 5-methyltetrahydrofolate concentrations (16) in this population.

Apart from folate given preconception (17), the impact of the timing of introduction of additional food rations in refugees— including multiple-micronutrient supplements (18) or micronutrients in vitamin supplements (19), which have the potential to affect fetal growth—is limited (3, 18, 20–26). The objective of this current analysis was to compare newborn outcomes (SGA, PTB, anthropometric measures, neonatal mortality) with the length of maternal exposure to a new ration that included micronutrient-fortified flour (MFF) in the Maela refugee camp, on a background of a pregnancy-specific ration and vitamin supplements (iron, folic acid, and thiamin) received at the ANC.

## METHODS

### Ethical approval

This study was registered in the German Clinical Trials Register (http://www.drks.de/DRKS00007736) and was approved by both the Ethics Committee of the Faculty of Tropical Medicine of Mahidol University, Thailand (TM-IRB 04/2004), and the Oxford Tropical Research Ethics Committee, University of Oxford, United Kingdom (OXTREC 009–04).

### Study design

**FIGURE 1:**
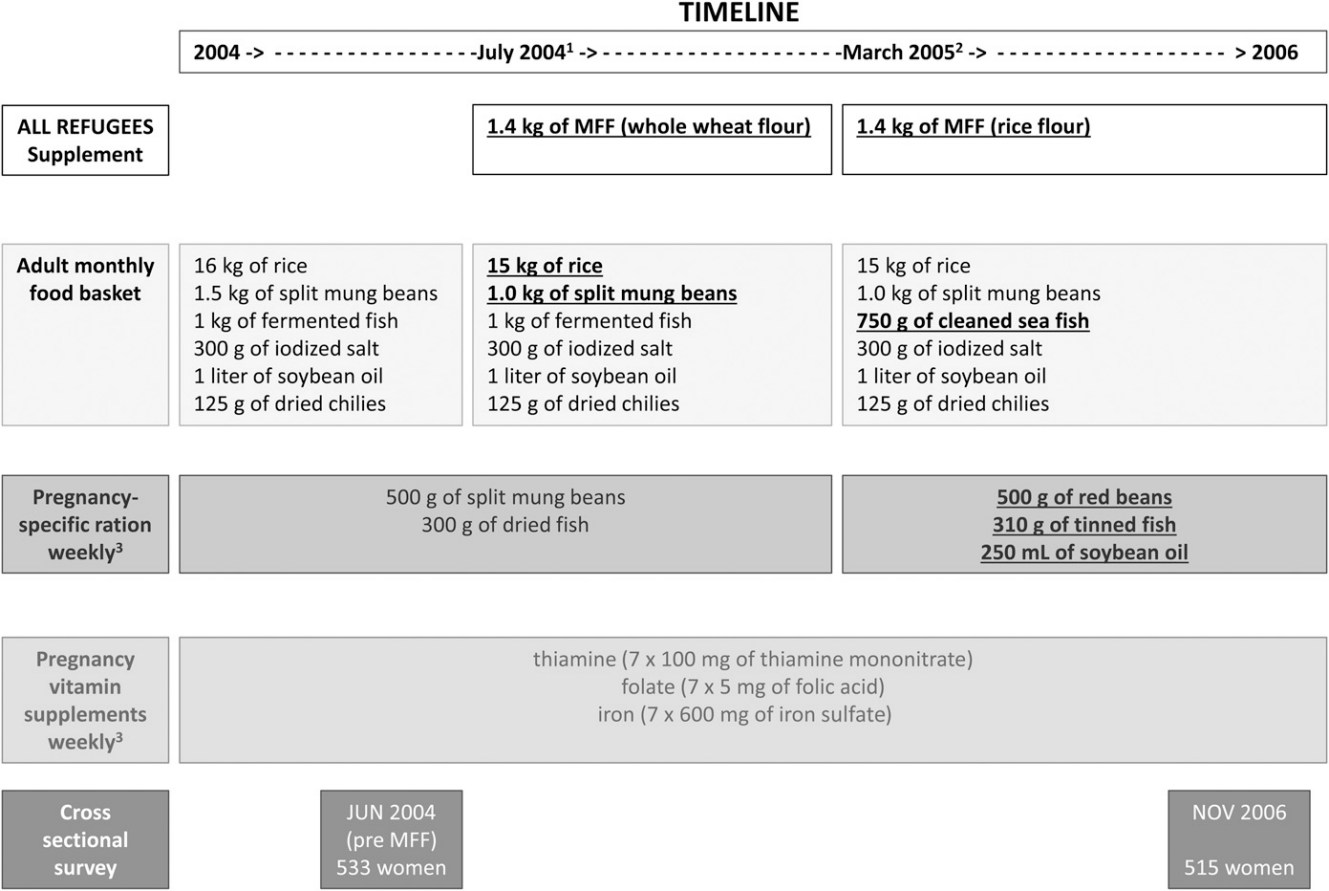
Refugee food basket and pregnancy-specific ration and vitamin supplements changes over time and timing of the cross-sectional surveys. Changes are indicated by bold type and underlined. ^1^Time the whole-wheat MFF was added to the refugee food basket. ^2^Time at which the whole-wheat MFF was replaced by rice flour MFF and when the modifications to the pregnancy-specific ration occurred. ^3^The duration of the weekly pregnancy-specific ration and pregnancy vitamin supplements was dependent on the first presentation to the antenatal clinic. MFF, micronutrient-fortified flour.

Two cross-sectional surveys were designed to capture the micronutrient status of the pregnant population attending antenatal care in the Maela refugee camp before and after MFF introduction in the refugee ration. Data from these 2 surveys with regard to micronutrient status, but not neonatal outcomes, have been published (16, 27, 28). The first survey took place in June 2004, 1 mo before adding whole-wheat MFF in the refugee food basket in July 2004 (**Figure 1**). The second cross-sectional survey took place in November 2006, 20 mo after replacing the whole-wheat MFF with rice MFF and modifying the pregnancy-specific ration. This ensured that, theoretically, pregnant women in the camp received a consistent ration containing MFF for ≥3 mo preconception. There were no major changes in the provision of antenatal or delivery care, in new refugee conflicts that could affect attendance or ration deliveries, or in general living conditions in the camp during this period.

### Setting

Thailand has sheltered refugees from Myanmar since 1986. The largest camp, Maela, 50 km north of Mae Sot, currently shelters ∼40,000 refugees. In Maela, the major provider of medical care has shifted from Médecins Sans Frontières to Première Urgence–Aide Médicale Internationale in 2002 and the American Refugee Committee in 2016, with the Shoklo Malaria Research Unit (SMRU) providing antenatal, birthing, and postnatal care services since 1996.

### Participants

The surveys were exhaustive and aimed to include all women if they attended SMRU ANC and had a viable pregnancy at the time of the cross-sectional survey. Women were excluded from the study if they had severe anemia, no receipt of the refugee ration, lived outside the camp, or planned to deliver elsewhere.

### SMRU antenatal care

Early and frequent antenatal attendance was encouraged by SMRU and Première Urgence–Aide Médicale Internationale staff. Weekly visits permit early detection and treatment of malaria until delivery, with intermittent preventive treatment not being available for highly drug-resistant *Plasmodium falciparum* (29). ANC attendance was voluntary. Some women could attend every week and others had less-regular visits. Unwell women were able to attend the clinic at any time.

Estimated gestational age (EGA) by ultrasound was obtained at the first ANC visit (30) as was maternal weight (measured to the nearest 0.5 kg) and height (nearest 1 mm). The following cutoffs indicating underweight and overweight for the Asian population were used: BMI (in kg/m^2^) <18.5 and ≥23.0, respectively (31). Pregnancy-specific vitamin supplements provided at each visit included thiamin (7 × 100 mg thiamin mononitrate), folate (7 × 5 mg folic acid), and iron (7 × 600 mg ferrous sulfate) (13, 32) (Figure 1). Service uptake was high, with the majority of women voluntarily giving birth with skilled birth attendants at the SMRU clinic (15).

### Refugee ration

A review of the ration provided to refugees under the auspices of The Border Consortium (http://www.theborderconsortium.org/) suggested that the ration was not adequate for long-term survival, especially in children (33). Hence, in 2004, the ration changed for all camp residents. Increases in vitamins and micronutrients were not matched by an increase in the estimated caloric intake (**Supplemental Table 1**).The main change was the addition of 1.4 kg whole-wheat MFF/mo for adult inhabitants of the Maela camp (28), together with a reduction in the ration of rice, split mung beans, and fish (Figure 1), which is referred to as the “new ration.” In March 2005, an additional minor change included the replacement of whole-wheat MFF with rice-based MFF for better acceptance by the refugees (Supplemental Table 1); and fermented fish was replaced by 750 g cleaned sea fish. Details of the proportional changes in daily total energy (calories) and micronutrients as well as the monthly adult food basket and pregnancy-specific ration are presented in Supplemental Table 1. With the monthly ration distribution, intrahousehold redistributions likely took place and households who could afford it could supplement the monthly food basket by purchasing seasonal fruit and vegetables (with no shortage during any period of the year) from the markets within the camp. Poultry in the camp were culled before the 2006 survey due to the Asian bird flu outbreak (H5N1) (34). The weekly supplementary food issued to pregnant women at the ANC, referred to as the pregnancy-specific ration, also changed: from 500 g split mung beans and 300 g dried fish in June 2004 to an additional weekly ration of soybean oil (250 mL) and the replacement of split mung beans and dried fish with red beans and tinned fish in March 2005 (Figure 1). In an effort to ensure that the MFF was used by recipients, The Border Consortium held information sessions with zone and section leaders and recipe demonstrations around the camp including at ANCs and provided simple recipe books to families.

### Birth outcomes

Every newborn was weighed with the use of an electronic scale (Seca 335, precision of 5 g) within the first hour if born in the ANC or when they presented to the ANC if born at home. Weight measured on days 2 and 3 (*n* = 112) was corrected for weight loss by 3.6% (95% CI: −3.9%, −3.4%) and by 4.4% (95% CI: −4.7%, −4.1%), respectively (35). Birth length (BL) was assessed on a measuring board (Seca 210, precision of 5 mm). A Seca 202 measuring tape (precision of 1 mm) was used to measure head circumference (HC). All of the measurements were performed by health workers trained and quality-controlled in SMRU clinics.

Miscarriage was defined as delivery before 28 wk of gestation. SGA was defined by international standards for birth weight (BW), BL, and HC below the 10th percentile for EGA of separate standards for male and female infants (8). The *z* scores for HC, BW, and BL were calculated by using the Intergrowth-21 *z-*score calculator (https://intergrowth21.tghn.org/articles/intergrowth-21st-fetal-growth-standards/) (8). Low BW included infants <2500 g, and PTB was defined as birth occurring before an EGA of 37 wk. Death within the first 28 d of life was recorded as a neonatal death.

### Statistical analysis

Groups from the 2 surveys were compared by using the chi-square or Fisher’s exact test for categorical variables and Student’s *t* test or Mann-Whitney *U* test, depending on the distribution of the data. For comparisons across trimesters, a test for trend across ordered groups was used. Adjusted *P* values were obtained by using regression modeling, and fit was assessed by using the Hosmer-Lemeshow goodness-of-fit test (logistic regression) or by visual inspection of the distribution of the residuals (linear regression). The risk of PTB and SGA was quantified by using logistic regression to obtain ORs with 95% CIs.

Different BMI measurements were used in the analysis due, in part, to the varying first visit of women to the ANC and also to obtain good model fit, and these are identified in the table footnotes (**Tables 1**–**4**). Comparisons of women who presented for their first ANC visit in the first trimester used BMI at admission (Tables 1 and 3); comparisons of women who presented later used BMI before birth (Table 2). For the final comparison, BMI was used as a dichotomous variable of underweight (yes or no) at any stage of pregnancy (Table 4).

**TABLE 1:** Effect of the length of exposure to a new ration in women with the first ANC visit in trimester 1 on birth outcomes in 2004^1^

	Period in which new ration commenced				*P*-trend	
Birth outcomes	Trimester 1 (*n* = 31)	Trimester 2 (*n* = 188)	Trimester 3 (*n* = 133)	Postpartum (*n* = 45)	Unadjusted^2^	Adjusted^3^
NNDs, *n* (%)	0	0	1 (0.75)	0	0.516	0.558^4^
PTBs, *n* (%)^5^	1 (3.2)	16 (8.5)	Not included	Not included	0.309	0.282
LBW, *n* (%)	4 (12.9)	24 (12.8)	18 (13.5)	12 (26.7)	0.075	0.107
SGA, *n* (%)	6 (19.4)	35/186 (18.8)	36/132 (27.3)	13 (28.9)	0.050	0.046*
LGA, *n* (%)	1 (3.2)	9/186 (4.8)	4/132 (3.0)	0	0.197	0.220
EGA, wk	39.0 ± 1.17	38.7 ± 1.69	38.9 ± 1.41	38.6 ± 1.10	0.427	0.604
BW, g	3042 ± 457	2977 ± 471	2896 ± 420	2779 ± 344	0.001*	0.008*
BL, cm	49.3 ± 1.85 (*n* = 29)	48.6 ± 2.49 (*n* = 162)	48.8 ± 1.93 (*n* = 88)	48.5 ± 1.76 (*n* = 42)	0.422	0.947
HC, cm	32.3 ± 1.61 (*n* = 29)	32.2 ± 1.64 (*n* = 162)	31.9 ± 1.35 (*n* = 88)	31.6 ± 1.22 (*n* = 42)	0.017*	0.029*

1 Values are means ± SDs unless otherwise indicated. *P < 0.05. ANC, antenatal clinic; BL, birth length; BW, birth weight; EGA, estimated gestational age; HC, head circumference; LBW, low birth weight; LGA, large for gestational age; NND, neonatal death; PTB, preterm birth; SGA, small for gestational age.

2 Test for trend.

3 Test for trend adjusted for weeks of pregnancy; ferrous sulfate, folic acid, and thiamin supplements; smoking (yes or no); gravidity (first vs. subsequent pregnancy); maternal age at admission; maternal BMI at admission; sex of infant; day of weighing infant (within first 24 h vs. after 24 h); malaria (yes or no); and hypertension (yes or no) (Supplemental Table 2).

4 Model adjusted for weeks of pregnancy; ferrous sulfate, folic acid, and thiamin supplements; maternal age at admission; and maternal BMI at admission only.

5 Only the first and second trimesters were analyzed for this outcome.

**TABLE 2:** Effect of the timing of exposure to the pregnancy-specific ration and vitamin supplements on a background of the preconception new ration by trimester at enrollment in an ANC on birth outcomes in 2006^1^

	Trimester in which pregnancy-specific ration and vitamin supplements commenced^2^		*P*	
Birth outcomes	Trimester 1 *(n* = 400)	Trimester 2 *(n* = 70)	Unadjusted^3^	Adjusted^4^
NNDs, *n* (%)	7 (1.8)	2 (2.9)	0.629	0.889
PTBs, *n* (%)	21 (5.3)	6 (8.6)	0.267	0.427
LBW, *n* (%)	35 (8.8)	6 (8.6)	0.961	0.678
SGA, *n* (%)	69/398 (17.3)	8 (11.4)	0.219	0.155
LGA, *n* (%)	16/398 (4.0)	3 (4.3)	0.917	0.932
EGA, wk	39.1 ± 1.52	39.3 ± 1.64	0.119	0.060
BW, g	3044 ± 461	3041 ± 420	0.735	0.866
BL, cm	49.2 ± 2.14 (*n* = 395)	48.9 ± 2.11	0.179	0.370
HC, cm	32.4 ± 1.41 (*n* = 395)	32.4 ± 1.47	0.663	0.730
*z* Score				
BW	−0.36 ± 0.99 (*n* = 398)	−0.42 ± 0.85	0.363	0.724
BL	0.14 ± 1.12 (*n* = 393)	−0.11 ± 1.00	0.070	0.123
HC	−1.11 ± 1.10 (*n* = 393)	−1.13 ± 1.01	0.934	0.866

1 Values are means ± SDs unless otherwise indicated. ANC, antenatal clinic; BL, birth length; BW, birth weight; EGA, estimated gestational age; HC, head circumference; LBW, low birth weight; LGA, large for gestational age; NND, neonatal death; PTB, preterm birth; SGA, small for gestational age.

2 Includes only women who presented to an ANC in the first or second trimester.

3 Chi-square or Fisher’s exact tests for categorical variables and Student’s *t* test or Mann-Whitney *U* test depending on the distribution of the data.

4 Regression analysis adjusted for smoking (yes or no), gravidity (first vs. subsequent pregnancy), maternal age at admission, maternal BMI before delivery, sex of infant, day of weighing infant (within first 24 h vs. after 24 h), malaria (yes or no), and hypertension (yes or no) (Supplemental Table 3).

**TABLE 3:** Impact of no ration compared with comprehensive ration exposure in women with the first ANC visit in trimester 1 on birth outcomes^1^

			*P*	
Birth outcomes	No ration: 2004 (*n* = 45)	Comprehensive ration: 2006 (*n* = 400)	Unadjusted^2^	Adjusted^3^
NNDs, *n* (%)	0	7 (1.78)	1.000	—
PTBs, *n* (%)	3 (6.7)	21 (5.3)	0.724	0.860
LBW, *n* (%)	1 (26.7)	35 (8.8)	<0.001*	<0.001*
SGA, *n* (%)	13 (28.9)	69/398 (17.3)	0.059	0.050
LGA, *n* (%)	0	16 (4.0)	0.390	—
EGA, wk	38.6 ± 1.10	39.1 ± 1.52	0.004*	0.006*
BW, g	2779 ± 344	3044 ± 461	<0.001*	<0.001*
BL, cm	48.5 ± 1.76 (*n* = 42)	49.2 ± 2.14 (*n* = 395)	0.016*	0.028*
HC, cm	31.6 ± 1.12 (*n* = 42)	32.4 ± 1.41 (*n* = 395)	<0.001*	0.001*
*z* Score				
BW	−0.88 ± 0.85	−0.36 ± 0.99 (*n* = 398)	<0.001*	0.001*
BL	−0.11 ± 0.99 (*n* = 42)	0.14 ± 1.12 (*n* = 393)	0.127	0.174
HC	−1.60 ± 0.88 (*n* = 42)	−1.11 ± 1.10 (*n* = 393)	<0.001*	0.003*

1 Values are means ± SDs unless otherwise indicated. *P < 0.05. ANC, antenatal clinic; BL, birth length; BW, birth weight; EGA, estimated gestational age; HC, head circumference; LBW, low birth weight; LGA, large for gestational age; NND, neonatal death; PTB, preterm birth; SGA, small for gestational age.

2 Chi-square or Fisher’s exact test for categorical variables and Student’s *t* test or Mann-Whitney *U* test depending on the distribution of the data.

3 Regression analysis adjusted for smoking (yes or no), gravidity (first vs. subsequent pregnancy), maternal age at admission, maternal BMI at admission, sex of infant, day of weighing infant (within first 24 h vs. after 24 h), malaria (yes or no), and hypertension (yes or no).

**TABLE 4:** Risk factors for preterm birth and small for gestational age^1^

	Small for gestational age (*n* = 983)		Preterm birth^2^ (*n* = 989)	
Risk factors	OR (95% CI)	*P*	OR (95% CI)	*P*
BMI (in kg/m^2^) <18.5^3^	2.51 (1.64, 3.83)	*<*0.001*	1.61 (0.81, 3.19)	0.177
Smoking	2.19 (1.51, 3.20)	*<*0.001*	3.34 (1.79, 6.21)	*<*0.001*
Primigravida	2.71 (1.70, 4.32)	*<*0.001*	2.39 (1.20, 4.75)	0.012*
Age	1.03 (0.99, 1.06)	0.110	0.92 (0.87, 0.98)	0.007*
Sex	1.04 (0.74, 1.46)	0.821	1.21 (0.70, 2.09)	0.494
Malaria	1.08 (0.55, 2.12)	0.825	1.05 (0.39, 2.83)	0.926
Hypertension	0.89 (0.35, 2.25)	0.802	2.58 (0.71, 9.37)	0.150
Trimester of first ANC^4^ visit	0.70 (0.45, 1.09)	0.115	1.14 (0.62, 2.07)	0.678
Study year	0.68 (0.49, 0.96)	0.026*	0.82 (0.48, 1.41)	0.478

1 ORs (95% CIs) were derived from adjusted logistic regression analysis. *P < 0.05.

2 There were no preterm births in women who presented for their first ANC visit in trimester 3; more than three-fourths of all preterm births occurred in women who presented in trimester 1 (77.6%; 52 of 67); therefore, all women were retained in the model.

3 BMI <18.5 at any time during pregnancy.

4 ANC, antenatal clinic.

## RESULTS

**FIGURE 2:**
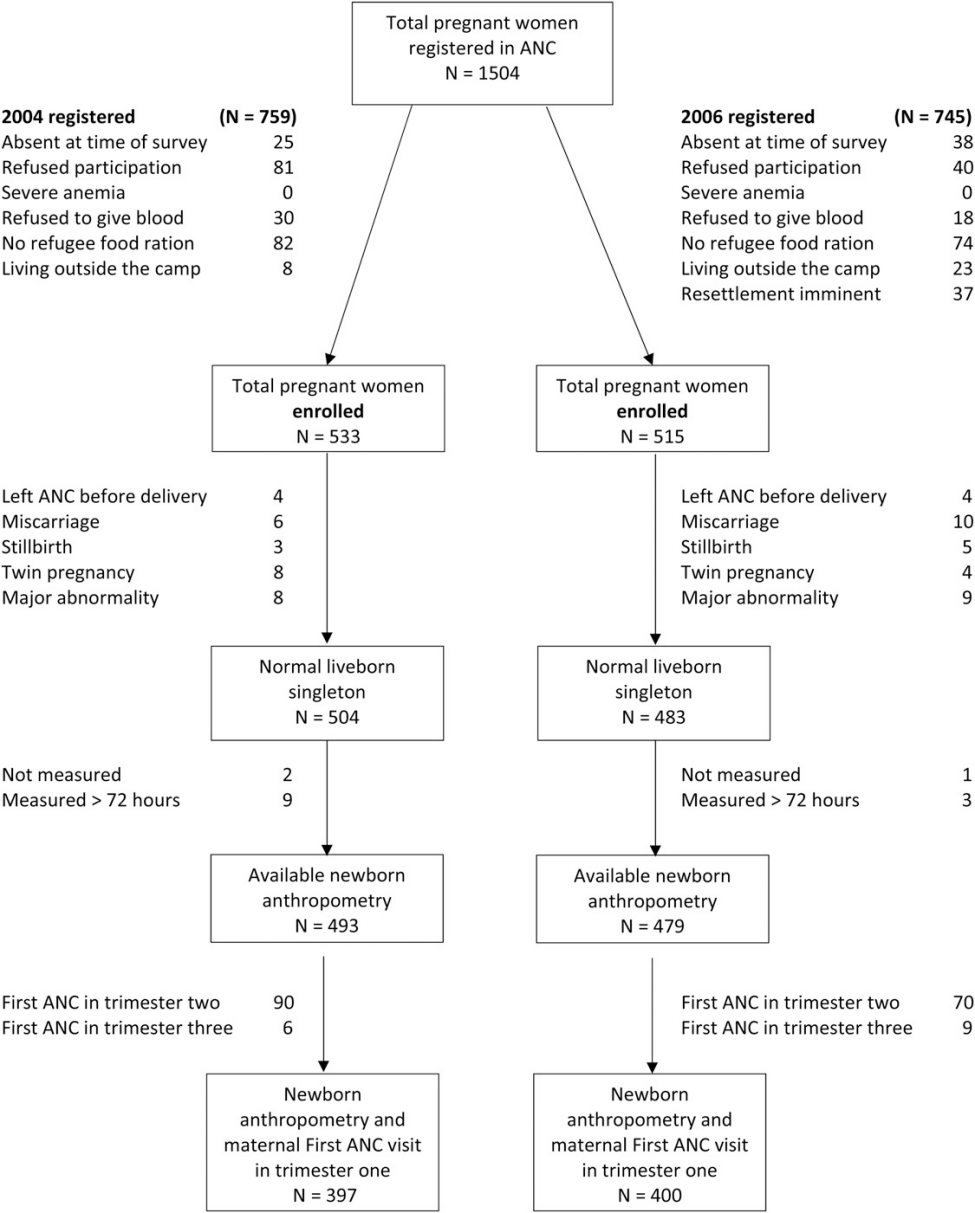
Study flowchart. ANC, antenatal clinic.

**Figure 2** shows that 1048 women were enrolled and 972 normal, live-born, singleton birth outcomes were available for analysis: 92.3% (493 of 533) and 93.0% (479 of 515) from 2004 and 2006, including 75.6% (397 of 533) and 78.4% (400 of 515) of newborns with the maternal first ANC visit in trimester 1, respectively. Exclusions resulted from the following: presentation of the newborn after 72 h (*n* = 12) or newborn was never measured (*n* = 3), miscarriage (*n* = 16), stillbirth (*n* = 8), twins (*n* = 12), or major congenital abnormality (*n* = 17). In the 2004 and 2006 surveys, the proportions of miscarriage [1.1% (6 of 533) and 1.9% (10 of 515); *P =* 0.285], stillbirth [1.9% (3 of 533) and 1.0% (5 of 515); *P =* 0.225], and major congenital abnormality [1.5% (8 of 533) and 1.7% (9 of 515)] did not differ significantly (*P =* 0.796).

### Effect of the timing of exposure of the new ration on birth outcomes in 2004

This section of analysis was confined to women who had presented for their first ANC visit in the first trimester, and demographic and birth outcome data were summarized by the gestation period (trimester and postpartum) of commencement of the new ration (**Supplemental Table 2**). Exposure to the new ration varied for these women, although exposure to the pregnancy-specific ration (mung beans and dried fish) and vitamin supplements commenced in trimester 1. With longer exposure to the new ration, the proportion of SGA was reduced and mean BW and HC increased significantly (Table 1). PTB involved a low number of newborns, and although the proportion of PTB was reduced, this was not significant. There were no changes in the proportion of large-for-gestational-age (LGA) infants.

**FIGURE 3:**
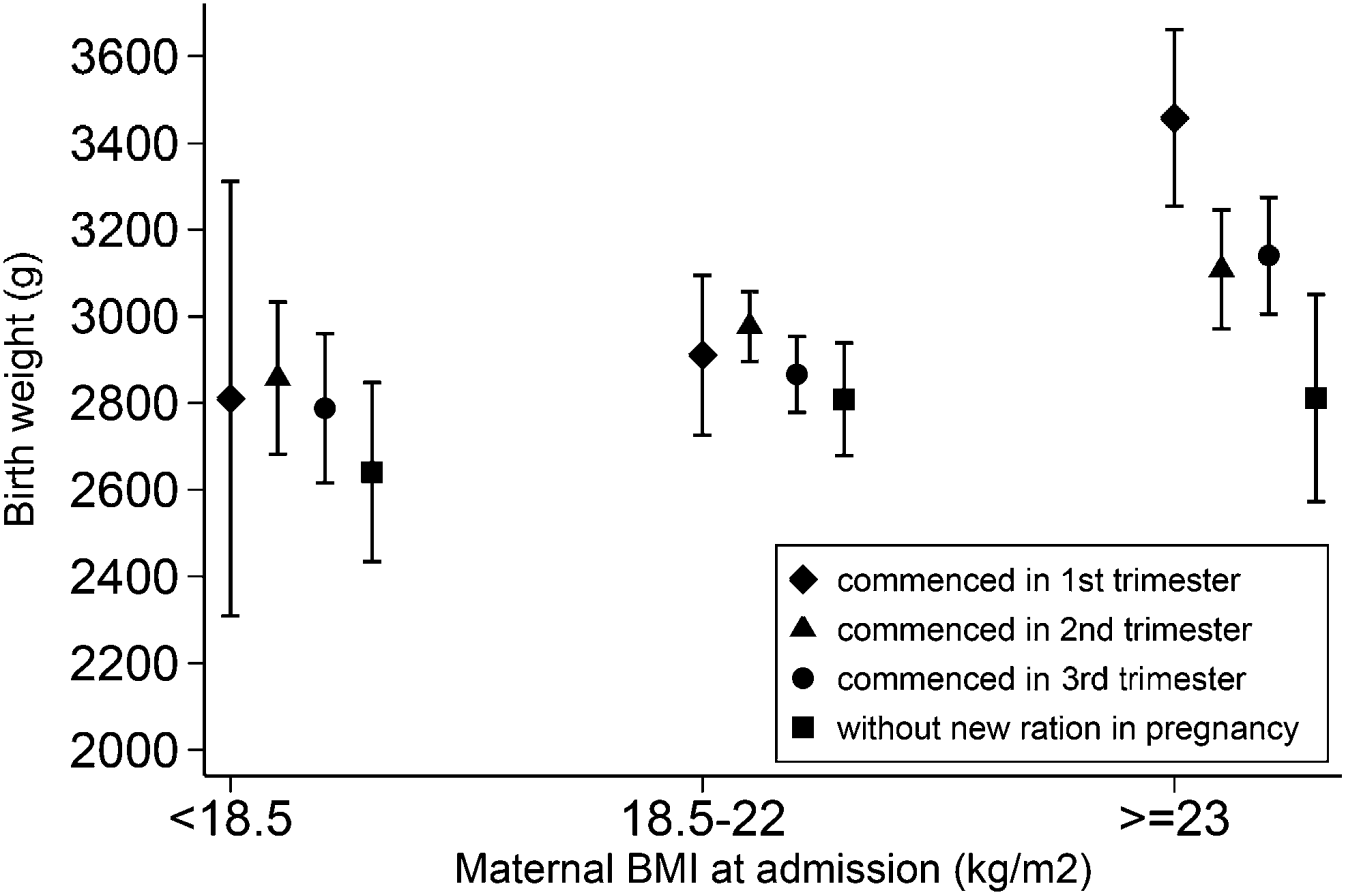
Birth weight by first-trimester BMI in women who commenced the new ration at different trimesters in pregnancy. Values are means (95% CIs).

The BW of newborns was plotted according to the BMI group of the mother and the period of gestation in which the new ration commenced (**Figure 3**). The trend for improved mean BW with earlier introduction of the new ration is shown in Figure 3 but appears to be more evident among overweight women.

### Effect of the timing of exposure to the pregnancy-specific ration and vitamin supplements on birth outcomes in 2006

The demographic and birth outcomes of women in the 2006 survey were compared according to the timing (trimester) of the first ANC visit, which coincided with starting the pregnancy-specific ration and vitamin supplements. All of these women had theoretically received the new ration preconception. There were only 9 women who presented for their first ANC visit in the third trimester, and therefore they were excluded from this part of the analysis. There were no neonatal deaths, PTBs, or low-BW or SGA infants among these late ANC attendees.

There were fewer weeks of intake of vitamin supplements in women who visited the ANC in the second than in the first trimester of pregnancy (**Supplemental Table 3**). Nevertheless, on a background intake of the preconception new ration, no significant difference was observed for birth outcome when women started the pregnancy-specific ration and vitamin supplements in first or second trimester (Table 2).

### Birth outcomes in 2004 and 2006

The birth outcomes of first-trimester attendees who delivered without receiving the new ration in 2004 and of nutritionally replete women (preconception new ration and pregnancy-specific ration and vitamin supplements from the first trimester) in 2006 were compared (**Supplemental Table 4**). Significant improvements in newborn outcomes in these 2 groups between 2004 and 2006 were observed, after adjustment for other factors affecting birth outcomes (Table 3): SGA decreased from 28.9% (13 of 45) to 17.3% (69 of 398) (adjusted *P =* 0.050), a reduction of 40.1% (95% CI: 34.7%, 45.9%), and LGA births remained low. Although there was a marked 38.2% (95% CI: 31.4%, 45.5%) reduction in underweight women on admission to the ANC between the 2 surveys, there was also an increase in the proportion of overweight women (11.5%; 95% CI: 7.8%, 16.7%) (Supplemental Table 4). The frequency of PTB was low, with a small, nonsignificant decrease between surveys: 6.7% (3 of 45) to 5.3% (21 of 400) (adjusted *P =* 0.860). Mean gestational age, BW, and HC and *z* scores for BW and HC were significantly higher in 2006 than in 2004 in both adjusted and unadjusted analyses, whereas BL increased, on average, by 0.7 cm (Table 3).

### Factors affecting SGA and PTB

The data for all women from both surveys were pooled for the regression analysis of risk factors for SGA and PTB among refugees (**Supplemental Table 5**). The risk of being born SGA significantly increased by maternal smoking, low maternal BMI (<18 at any time in pregnancy), and primigravid status and decreased by being in the 2006 survey group. The risk of PTB significantly increased by maternal smoking and primigravid status and decreased with increasing maternal age. There was no effect of survey group on the risk of PTB (Table 4).

## DISCUSSION

This study describes a reduction in SGA in association with changes in the refugee ration in a pregnant population nutritionally dependent on the refugee food basket. The strengths of the study include the high proportion of women presenting to an ANC in the first trimester (76%) and later giving birth with skilled birth attendants (>75%), accurate gestational age assessment with ultrasound, and multiple antenatal visits (average of 25 visits/woman). Because this was an assessment of the systematic rollout of a changed ration to the population, there were limitations with the trial design. Women were enrolled at the gestational period they were in at the time of each cross-sectional survey, but full access to antenatal records was possible from the first ANC visit. The new ration consumption was assumed rather than confirmed, although its uptake was considered adequate in surveys conducted by The Border Consortium, independently from the SMRU (http://www.theborderconsortium.org/media/10769/2007-nutrition-programme-outline-1.pdf).

Limitations of this analysis resulted from the need to be cognizant of >1 change made to the refugee ration. The general food basket caloric content was reduced in 2004, but certain micronutrients via MFF increased, particularly vitamin A, thiamin, riboflavin, vitamin C, iron, and calcium (Supplemental Table 1). Less than 1 y later, the type of MMF changed from whole wheat to rice based. In 2006, there was an adjustment of the pregnancy-specific ration, principally with the addition of oil to increase calories required in pregnancy. The significant linear trend for reduced SGA with longer exposure to the new ration was observed in 2004, with the effect (if any) of the pregnancy-specific ration and vitamin supplements controlled for by including only women enrolled in the first trimester.

The positive impact on SGA in 2004 was associated with significant increases (>50%) in vitamin A, thiamin, nicotinamide, riboflavin, vitamin C, iron, and calcium, and a small reduction in folic acid (Supplemental Table 1). A higher folic acid content in the MFF may have been an important omission given the reported benefits on birth outcome from this vitamin (17, 22, 36). In 2006, SGA was not associated with pregnancy-specific ration and vitamin supplements, indicating that they may be less important in the presence of an adequate preconception ration. In addition, 74% of women presented to the ANC in the first trimester (only 9 in trimester 3), so there was reduced power to show a difference between trimesters.

In 2004, there was a limited number of women who delivered just before the new ration distribution (*n* = 45 with no ration) and who attended the ANC from trimester 1, and these were compared with a much larger group from 2006 (*n* = 400 with the new ration preconception and during the entire pregnancy). A 40.1% (95% CI: 34.7%, 45.9%) reduction in SGA was observed, but cautious interpretation is required due to study design limitations; the 2004 group is small compared with the 2006 group and the reduction may be due to other non–study-related changes that occurred between 2004 and 2006 that were not controlled for.

Each comparison suggested reduced PTBs with longer exposure to the new ration; however, the overall numbers were small and nonsignificant. No conclusions with regard to the outcomes of miscarriage, stillbirth, and neonatal deaths were possible due to the small number of these events; however, there was no suggestion of negative effects of the new ration on these outcomes (Figure 2). There was no significant increase in the proportion of LGA with longer exposure to supplements.

The importance of maternal undernutrition on newborn outcomes has been recognized for decades (37). The factors associated with SGA in this study (i.e., maternal underweight, smoking, and being primigravid) are consistent with previous reports and support the robustness of the data (11, 38). There were no other significant changes in refugee camp life during this period, and the reduction in SGA was still observed on the background of the Asian bird flu outbreak (H5N1) and poultry culling before the 2006 survey (34).The more overt effect on mean BW in overweight women (Figure 3) is of concern in terms of nutrition transition and diseases such as diabetes and hypertension, which complicate pregnancy care, especially in resource-limited settings. Other studies observed the failure to increase mean BW in underweight women (18, 39). In this community, the effects on mean BW appeared to be similar for underweight and normal-weight women, which could relate to compliance or household food redistribution. Underweight women appear to be a particularly challenging target group. Improved general refugee rations would be key to reducing the proportion of women who fall into this category, as shown here.

In conclusion, data from refugee populations are difficult to obtain, so the positive association of reduced SGA with the changed ration of the refugee food basket (predominantly introduction of MFF) is encouraging. In nutritionally compromised groups it is possible to positively influence birth outcomes with appropriately timed food rations.

## Footnotes

9 Abbreviations used: ANC, antenatal clinic; BL, birth length; BW, birth weight; EGA, estimated gestational age; HC, head circumference; LGA, large for gestational age; MFF, micronutrient-fortified flour; PTB, preterm birth; SGA, small for gestational age; SMRU, Shoklo Malaria Research Unit.
